# Advanced Magnetic Resonance Imaging Modalities for Breast Cancer Diagnosis: An Overview of Recent Findings and Perspectives

**DOI:** 10.3390/diagnostics12112741

**Published:** 2022-11-09

**Authors:** Daryoush Shahbazi-Gahrouei, Fahimeh Aminolroayaei, Hamide Nematollahi, Mohammad Ghaderian, Sogand Shahbazi Gahrouei

**Affiliations:** 1 Department of Medical Physics, School of Medicine, Isfahan University of Medical Sciences, Isfahan 8174673461, Iran; 2 Department of Management, School of Humanities, Najafabad Branch, Islamic Azad University, Najafabad 8514143131, Iran

**Keywords:** diagnosis, MRI, diffusion tensor imaging, diffusion-weighted imaging, breast cancer

## Abstract

Breast cancer is the most prevalent cancer among women and the leading cause of death. Diffusion-weighted imaging (DWI) and diffusion tensor imaging (DTI) are advanced magnetic resonance imaging (MRI) procedures that are widely used in the diagnostic and treatment evaluation of breast cancer. This review article describes the characteristics of new MRI methods and reviews recent findings on breast cancer diagnosis. This review study was performed on the literature sourced from scientific citation websites such as Google Scholar, PubMed, and Web of Science until July 2021. All relevant works published on the mentioned scientific citation websites were investigated. Because of the propensity of malignancies to limit diffusion, DWI can improve MRI diagnostic specificity. Diffusion tensor imaging gives additional information about diffusion directionality and anisotropy over traditional DWI. Recent findings showed that DWI and DTI and their characteristics may facilitate earlier and more accurate diagnosis, followed by better treatment. Overall, with the development of instruments and novel MRI modalities, it may be possible to diagnose breast cancer more effectively in the early stages.

## 1. Introduction

Breast cancer is the most prevalent cancer in women [1]. Magnetic resonance imaging (MRI) has been drastically utilized to detect breast cancer due to its high contrast resolution. In the past few decades, developments in instruments, contrast agents, and modalities have brought MRI into a new era of breast cancer diagnosis [1,2]. MR imaging modalities include T_2_-weighted imaging, dynamic contrast-enhanced imaging (DCE-MRI), diffusion-weighted imaging (DWI), diffusion tensor imaging (DTI), and MR spectroscopy (MRS). Although routine MRI has a high sensitivity (80–100%) among these techniques, it lacks characterization specificity for breast cancer [3]. Even though there is still a substantial link between DCE-MRI and tumor vascular structures, there is no confirmation that this approach is connected with tumor cellular proliferation [4]. Moreover, DCE-MRI specificity can be as low as 37% or as high as 97%. However, the development of DCE-MRI involves higher costs than other techniques, and it cannot be utilized with contrast agents for patients with renal dysfunction [5]. Therefore, multimodality imaging may cover some limitations of MRI models [6]. Diffusion-weighted imaging is helpful to measure the portability of water particles diffusing in tissue. Its technological benefits include a fast acquisition period (usually three min), vast accessibility to various commercial scanners, and no requirement for administering contrast agents [7]. On the other hand, its limitation is sensitivity to artifacts such as T_2_ shine through, T_2_ blackout, ghosting, blurring, and distortions [8].

Generally, diffusion is measured qualitatively on trace images and quantitatively by the apparent diffusion coefficient (ADC), a parameter that can be used to map the limited diffusion of tissues on the trace image and hypointense [8]. During post-processing, ADC is calculated using at least two distinct b values. The literature indicates that the ADC value is the slope of a line superimposed on the relative signal intensity (y-axis) logarithm plot versus the b value (x-axis). An ADC map refers to the ultimate image with different ADC values determined for each image pixel. It is noted that ADC maps contain weak anatomical details and can be studied alongside other MRI images [9]. Most breast tumors (75–80%) are positive for estrogen receptors, and ~75% of them are positive for progesterone receptors [10]. Variations in ADC values according to estrogen or progesterone receptor expression have been documented in different studies [11,12,13]. Tumors that are positive for estrogen or progesterone receptors appear to have fewer ADC values than those which are negative [14]. The capacity of ADC measurements to distinguish between benign and malignant non-mass-like enhancement lesions is limited. Therefore, for non-mass-like enhancement lesions relative to mass lesions, a higher ADC value cutoff might be appropriate [15]. DWI is usually carried out before the administration of contrast. Additionally, it can be conducted following a contrast decrease in the ADC value [3]. According to studies, when diffusion is performed after DCE-MRI, a contrast agent may reduce the ADC value. However, after contrast administration with fat suppression (STIR technique), no critical effect has been demonstrated [16,17]. A DCE-MRI protocol exclusive to one non-contrast phase and one post-contrast phase has also received consideration to evaluate breast cancer [18,19]. Numerous research studies have shown that the detection rate of a DWI-based abbreviated unenhanced MRI protocol is comparable to a contrast-enhanced (CE) abbreviated protocol [20,21,22].

Water diffuses freely and isotropically in most conditions. When impenetrable barriers are encountered, it becomes anisotropic with an ellipsoidal shape [23], with rapid, unrestricted diffusion by the boundaries and slower, constrained diffusion vertical to the boundaries, based on the cell size and density. Diffusion in the ductal/glandular system is rapid due to the breast’s high water content and low cell density. When cancer cells obstruct the ducts and lobules, water transport is restricted, decreasing diffusion coefficients in all directions [24]. Although DWI does not detect this ellipsoidal shape, DTI has overcome this restriction. Theoretically, in DTI, at least six independent diffusion gradients measurements accompanied by six non-collinear directions are needed to evaluate all diffusion tensor elements [23]. At present, the most commonly used invariant anisotropy test is fractional anisotropy (FA) [25]. However, the FA has defined no complete tensor form or distribution because different combinations of eigenvalues will produce the same FA values.

In diffusion, forms of pulse sequences include spin-echo, stimulated-echo, and steady-state free precession (SSFP). A spin-echo pulse sequence is the most common method for producing MR imaging sensitive to diffusion. The diffusion-weighted stimulated echo sequence is important for tissues with short T_2_ relaxation times (liver). It can be combined with reading techniques, such as echo-planar imaging (EPI) or spiral imaging. To mitigate the effects of subject motion and maintain a high signal-to-noise ratio (SNR), the EPI method is used to achieve fast image acquisition. Echo-planar imaging is vulnerable to artifacts such as ghosting, chemical shift, and distortions [26]. It has been shown that parallel imaging techniques to reduce echo train lengths minimize susceptibility-related EPI artifacts in DWI and improve image quality, particularly at a field strength of 3 T [27]. Echo-planar imaging, nevertheless, is constrained by noise and typically uses thicker slices compared to CE-T_1_ imaging [3]. Theoretically, stimulated echoes have just half the signal compared to spin echoes. Furthermore, rapid image formation is possible regarding the very short imaging of T_R_ SSFP, which has high sensitivity to flow and diffusion [28].

This review aims to describe advanced magnetic resonance imaging modalities and recent findings in the literature on the diagnostics of breast cancer in the early stages.

## 2. DWI in the Field of Diagnostic Breast Cancer

Diffusion (Brownian motion) is a term that refers to the arbitrary, tiny development of water and other small molecules due to thermal excitation. When diffusion is similar in every direction, it is isotropic and is denoted by a single diffusion coefficient (D) as follows:

$$D_{\mathrm{isotropic}}=[\begin{array}{ccc} D_{\mathrm{xx}} & 0 & 0\\ 0 & D_{\mathrm{yy}} & 0\\ 0 & 0 & D_{\mathrm{zz}} \end{array}]$$

where ($D_{\mathrm{xx}}$ = $D_{\mathrm{yy}}$ = $D_{\mathrm{zz}}$ = D).

The signal intensities in DWI are:

$$s_{\mathrm{DWI}}=\sqrt[{3}]{s_{x}s_{y}s_{z}}=s_{0}\;\times \;e^{-b(D_{\mathrm{xx}}+D_{\mathrm{yy}}+D_{\mathrm{zz})/3}}=s_{0}\;\times \;e^{-b\left(\mathrm{trace}\right)/3}=s_{0}\;\times \;e^{-b.\mathrm{ADC}}$$

where diffusion trace is expressed as $D_{\mathrm{xx}}+D_{\mathrm{yy}}+D_{\mathrm{zz}}$ and average trace is ADC.

It is noteworthy that ADC in the breast (fibro-glandular tissue) is equal to 2.37 × 10^−3^ mm^2^/s [29]. DWI can be obtained by placing two additional diffusion-sensitizing gradients on each side of a spin-echo sequence’s 180° radiofrequency (RF) pulse. The diffusion weighting’s magnitude is achieved as follows:

$$b=\gamma^{2}G^{2}\delta^{2}\;(\Delta -\frac{\delta }{3})$$

where γ is the gyromagnetic ratio, G is the magnitude of the diffusion sensitizing gradients, δ is the temporal duration of each gradient, and Δ is the time interval between the application of the gradients. 

The ADC value is a function of the chosen b values. Thus, when greater b values are used, the ADC values become smaller, and the specificity of DWI can be increased by higher b values [13]. Images with a high b value exhibit a decreased SNR and higher image distortion [30].

**Figure 1:**
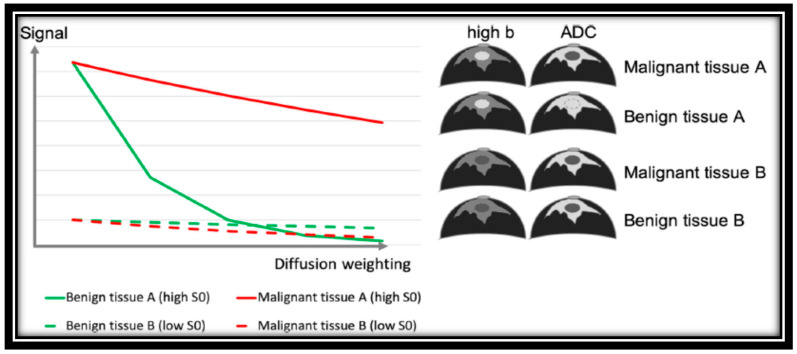
Signal decay of benign and malignant breast tissue in diffusion weighting imaging depending on baseline T_2_ signal [33]. “Reprinted with permission from Ref. [33]. 2020, springer”. More details on “Copyright and Licensing” are available via the following link: https://link.springer.com/article/10.1007/s00330-019-06510-3.

In a study, Dorrius et al. evaluated the b value’s effect and the contrast’s pre-admission on the diagnostic accuracy using 1.5 T for breast DWI. According to their research, b = 0 accompanied by 1000 s/mm^2^ is suggested to distinguish between normal and cancer tissues when time constraints allow only diffusion images obtained at a couple of amounts of b [16]; however, high b-value DWI accompanied by conventional MRI sequences can facilitate the diagnosis closely to DCE-MRI for breast cancer detection [31]. In many studies, it has been hypothetically shown that the ideal pair amounts of b to alleviate noise effects are in the range of 0 to 1000 s/mm^2^ in the brain (with moderately slow diffusion), while 0 and 800 s/mm^2^ are ideal in most low-water-content tissue such as breast [32,33]. Signal decay varies according to the baseline T_2_ signal in benign and malignant breast cancer, as indicated in Figure 1.

Diffusion-weighted imaging signal is generally T_1_- and T_2_-weighted, and higher-water-content lesions may have a high brilliance on DWI pictures; however, this is because of their exceptionally high T_2_ signal and is not associated with diffusion (T_2_ shine-through effect). One way to deal with this issue is to measure the ADC, which relies solely on diffusion [34]. Moreover, increasing the number of excitations in a fast DWI protocol has no diagnostic value according to Mori et al. They found no meaningful differences between one and four excitations in terms of lesion detectability or mean and minimum ADC value [35].

The common readout for breast DWI is single-shot EPI sequences, and readout segmented multi-shot EPI sequences. Echo planar imaging sequences are fast and insensitive to move, but they are vulnerable to geometric distortions that are most noticeable in the direction of phase encoding, leading to lower precision of quantification [36]. Moreover, EPI undergoes blurring due to T_2_* decay during readout. To decrease distortions, using some approaches such as high receiver bandwidth, parallel imaging, readout-segmented multi-shot EPI sequences [37], combined EPI acquisitions with integrated dynamic shimming [38], and reduced field of view (rFOV) [39] can be helpful.

In parallel imaging, the k-space lines are omitted, and the distance between them is increased.

Parallel imaging is widely used at a magnetic field strength of 3 Tesla since susceptibility effects are increased at greater field strengths. However, applying them at magnetic field strength 1.5 Tesla provides other advantages, such as increasing the number of slices for the same T_R_ and decreasing acquisition time. Although parallel imaging can reduce artifacts by decreasing the length of the echo train and hence reducing the T_2_* blurring, it is constrained by the hardware of the RF coil [40]. 

In rFOV, sharp images of a target region can be obtained by decreasing the matrix size, resulting in reduced susceptibility artifacts and higher spatial resolution, with the tradeoff of increased acquisition time, compared to single-shot EPI DWI. The depiction of lesion boundaries and heterogeneity partitioned from the encompassing fibro-glandular or adipose tissue can be troublesome with conventional EPI [7]. However, the latest research on breast imaging with rFOV DWI has demonstrated that the lesion visibility is increased, image quality is improved, and high resolution is obtained in comparison to images acquired with conventional bilateral DWI, representing them as a viable alternative of DCE-MRI in breast cancer patients [41].

By obtaining k-space in some segments, multi-shot methods often aim to decrease the length of the echo train. In this regard, Baxter et al. (2019) compared multi-shot DWI using multiplexed sensitivity encoding with conventional single-shot EPI-DWI. According to their study, ADC indicates a low variation coefficient (<2%) between single-shot EPI-DWI and multi-shot segmented EPI technique (multiplexed sensitivity encoding (MUSE-DWI) permutations), which was obtained by using the phantom. ADC values determined by MUSE were significantly lower in malignant lesions than single-shot EPI. Their study compared ADC and normalized ADC = ($\frac{{\mathrm{ADC}}_{\mathrm{lesion}}}{{\mathrm{ADC}}_{\mathrm{tissue}}}$) variations using the phantom variation coefficient and a paired t-test on the studied patients. Their result showed that normalized ADC values were not significantly different. They also stated that the quality of DWI breast images obtained by MUSE could be improved in comparison to single-shot EPI [40].

## 3. Comparison of DWI with Other Modalities

Generally, breast cancer can be examined with various techniques such as ultrasound, mammography, perfusion, and diffusion. According to many cohort and systematic studies, the sensitivity of MRI is higher than mammography, ultrasound, and a combination of ultrasound and mammography [42,43,44,45,46].

Many studies have shown that MR imaging has 91–100% sensitivity, and the specificity of DCE-MRI is about 37–97% [43,47]. Dynamic contrast enhancement MRI has disadvantages, such as a high cost, and cannot be applied to renal dysfunction patients. Based on the literature, among these techniques, DWI helps distinguish between benign and malignant breast lesions [48,49].

**Figure 2:**
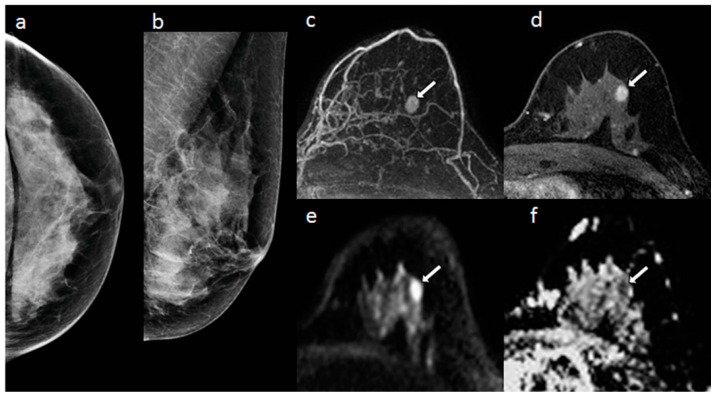
A 58-year old woman with dense breasts and invasive ductal carcinoma. (**a**) X-ray mammogram CC view and (**b**) MLO view, (**c**) DCE maximum intensity projection, (**d**) axial T_1_-weighted fat saturated DCE-MRI, (**e**) axial DWI, and (**f**) ADC map [5]. “Reprinted with permission from Ref. [5]. 2011, springer”. More details on “Copyright and Licensing” are available via the following link: https://link.springer.com/article/10.1007/s00330-010-1890-8. White arrows indicated lesion in the images.

In a study, Yabuuchi et al. [5] compared mammography, DCE-MRI, and a combination of diffusion-weighted and T_2_-weighted imaging (DWI + T_2_WI) to detect non-palpable breast cancer in asymptomatic women. As indicated in Figure 2, the lesion was not visible on two-view mammography. The mass was enhanced on post-contrast MRI, easily identified on MIP. The mass appears to have a high signal on DWI and low ADC on DWI and ADC maps. According to their report, DWI + T_2_WI showed a higher area under the curve (AUC) (AUC = 0.73; sensitivity = 50%) than mammography alone (AUC = 0.64; sensitivity = 40%), but lower than DCE-MRI (AUC = 0.93; sensitivity = 86%). Moreover, a combination of mammography and DWI + T_2_WI showed greater sensitivity (69%) than mammography alone (40%). Having said that, false-positive outcomes using MRI images in high-risk lesions are substantially different from false-positive findings by mammography in low-risk lesions, according to Kuhl et al. [50].

Zhang et al. conducted a study to determine which DCE-MRI, DWI, and T_2_-weighted image descriptors are most closely related to breast cancer diagnosis. Their study set the b value to 50 and 850 s/mm^2^. On DWI, malignant lesions exhibited a significantly lower average ADC mean (0.90 × 10^−3^ mm^2^/s) than benign lesions (1.43 × 10^−3^ mm^2^/s). They showed that DCE-MRI and DWI quantitative and qualitative variables are included in a multi-parametric MRI modality for breast cancer diagnosis. Indeed, they noted that models using the American College of Radiology (ACR) provide high diagnostic accuracy. Breast Imaging Reporting and Data System (BI-RADS) descriptors of margins and enhancement kinetics on DCE-MRI and ADC mean (either with DWI using a cutoff value or as a continuous variable) are mainly connected with a breast cancer diagnosis. Conventional T_2_-weighted imaging did not remarkably contribute to breast cancer diagnosis [51].

In 2019, Egnell et al. conducted a study to determine how stromal collagen fibers correlate with in vivo DWI. They used a high b value scheme to assess the association between collagen content and ADC and the signal fractions of the bi-exponential model. According to their study, collagen content is associated with the quick signal fraction and ADC values. They also found that the cellular content was inversely associated with the short signal fraction and ADC and varied between malignant, benign, and normal [52].

## 4. Different Models in DWI

As indicated in the literature, numerous models are available for extracting quantitative characteristics from images such as mono-exponential, intravoxel incoherent motion (IVIM) or bi-exponential, diffusion kurtosis imaging (DKI), stretched-exponential, Padé exponent (PE), statistical, and fractional-order calculus.

For instance, mono-exponential is the simplest model, often helpful if diffusion is analyzed on a voxel level [53]. The equation of mono-exponential is as follows:

$$S_{b}=S_{0}\;\times \;e^{-b.\mathrm{ADC}}$$

where $S_{b}$ is the signal intensity with b value, and ${\;S}_{0}$ is the signal intensity with b = 0. 

The signal for the bi-exponential IVIM model is given by:

$$S_{b}=S_{0}\;(1-f).e^{-b.D}+f.e^{-b\left(D+D^{*}\right)}$$

where D is true diffusivity, f is Perfusion fraction, and D* is pseudo-diffusivity.

In this model, which incorporates both diffusion and perfusion, f can be calculated as follows:

$$F=\frac{S\left(b\;=0\right)-{\;S}_{\mathrm{int}}}{S\left(b\;=0\right)}$$

where S_int_ can be assessed by a mono-exponential fit applied to the high b value range.

The equation of diffusion kurtosis is:

$$S=S_{0}\;\exp\;(-b.D_{K}+\frac{1}{6}b^{2}D_{K}^{2}.K)$$

where K is the kurtosis coefficient and D_K_ is the kurtosis corrected diffusion coefficient.

Anisotropic Gaussian diffusion is assumed by both the mono-exponential and IVIM models. Meanwhile, non-Gaussian diffusion can be considered by the kurtosis model.

**Figure 3:**
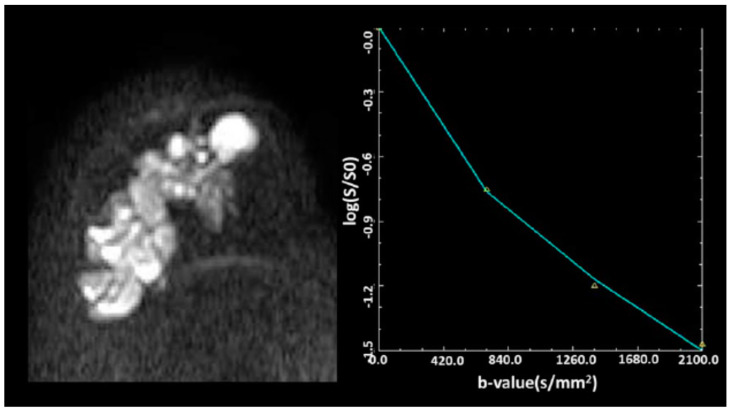
At the b value of 700–2100 s/mm^2^, the signal decay is slower, indicating a multi-exponential signal decay pattern of breast carcinoma [54]. Reprinted with permission from Ref. [54]. 2018, Wiley Online Library”. More details on “Copyright and Licensing” are available via the following link: https://onlinelibrary.wiley.com/doi/abs/10.1002/jmri.25904.

The signal of the stretched-exponential model is as follows:

$$S_{b}=S_{0}\;\times \;e^{-{\left(b.\mathrm{DDC}\right)}^{\alpha }}$$

where DDC (distributed diffusion coefficient) is a measure of signal decay rate with *b* values and a is the heterogeneity index between 0 and 1. Figure 3 provides an example of a fitting by the DDC model.

The equation of the Padé exponent (PE) is given by:

$$S=S_{o}.e^{\frac{-D_{p}.b}{1+\delta_{p}.b}}$$

where δ_p_ is quantified non-Gaussianity and D_p_ is diffusivity.

The signal of the statistical model is obtained by the following equation:

$$S=S_{o}.\frac{1+\varphi (\frac{{\mathrm{ADC}}_{\mathrm{peak}}}{\sigma \sqrt{2}}-\frac{b_{\sigma }}{\sqrt{2}})}{1+\varphi (\frac{{\mathrm{ADC}}_{\mathrm{peak}}}{\sigma \sqrt{2}})}e^{-b.{\mathrm{ADC}}_{\mathrm{peak}}+\frac{1}{2}b^{2}\sigma^{2}}$$

The equation of fractional order calculus is as:

$$S=S_{0}.e^{-{\left(b^{*}.D^{*}\right)}^{\beta }}$$

$$b^{*}=({\gamma G}_{d}\delta {)}^{2}\left(\Delta -\frac{2\beta -1}{2\beta +1}\delta \right)$$

$$D^{*}=D^{\frac{1}{\beta }}\mu^{2\left(1-\frac{1}{\beta }\right)}{\left(\Delta -\frac{2\beta -1}{2\beta +1}\delta \right)}^{\frac{1}{\beta }-1}$$

where G_d_ is the diffusion gradient amplitude, δ is the diffusion gradient pulse lobe duration, Δ is diffusion gradient pulse separation, D is the diffusion coefficient, b is the fractional-order derivative in space, and µ is a spatial parameter.

Igor Vidić et al. assessed different non-Gaussian representations of DWI signals for benign and malignant breast lesions in the b range of 200 to 3000 s/mm^2^. According to their study, diffusion signal models provided parameters with a high area under the curve (AUC > 0.9) for classifying benign and malignant lesions. In their reports, the highest AUC of 0.99 was achieved for f (bi-exponential), K (kurtosis), and 0.989 for D (fractional calculus). Additionally, non-Gaussian representations are required for fitting the DWI curve at high b values in breast lesions. Moreover, the single voxel analysis showed that the SNR provided high classification accuracy for the statistical and fractional calculus diffusion model. Meanwhile, the other non-Gaussian representations gave lower classification accuracy than the mono-exponential model [55].

Bedair et al. evaluated the response of breast tumors to neoadjuvant chemotherapy at a field strength of 3 T using mono-exponential, bi-exponential, and stretched-exponential models. Mean diffusion coefficients at pretreatment revealed substantial variations between the analyzed groups in their study. The percentage rise in ADC and DDC had substantial disparities between responders and non-responders, and stretched-exponential parameters showed excellent repeatability. They also showed that DWI is sensitive to early treatment changes in breast cancer utilizing non-mono-exponential models, and the stretched-exponential model can be used to monitor such changes [56].

In another work, Suo et al. examined breast lesion differentiation using mono-exponential, bi-exponential, stretched-exponential, and kurtosis models. All diffusion measurements showed substantial variations in their findings, except for mean diffusivity between benign and malignant lesions. Moreover, no significant variations between ADC and non-mono-exponential diffusion parameters were observed in areas under the ROC curves, except for Df (bi-exponential) and α (stretched-exponential), the AUCs of which were substantially lower than the ADC AUC for differentiating benign from malignant lesions. Df was strongly correlated with lymph node metastasis and Ki-67 expression in patients with invasive breast cancer. Moreover, ADC, Ds (bi-exponential), f (bi-exponential), DDC (stretched-exponential), and mean diffusivity were highly correlated with estrogen receptor status. Overall, they claimed that multi-parametric DWI is associated with breast lesions’ pathological outcomes and prognostic factors [57].

## 5. DWI in Treatment Evaluation of Breast Cancer

There are many uses for treatment planning in DWI that can help detect and characterize tumors and predict and evaluate therapy response in malignancies, wherein hyper-cellular metastases and fibrosis-restricted diffusion is mainly observed [9]. It is specified that tumors with a more significant mean ADC pretreatment are more likely to be necrotic [58], and may therefore include more hypoxic areas. Additionally, diffusion continues to increase during radiotherapy because of cell membrane destruction and treatment-induced cell death [59,60]. 

Mammography, sonography, and MRI can be used to check the size changes after neoadjuvant chemotherapy (NAC). MRI may be an effective strategy for studying the impact of NAC, and it may be accommodating to choose the extent of surgery. In this regard, the sensitivity of MRI in the initial staging of breast cancer and detecting the residual cancerous tissues following neoadjuvant chemotherapy was reported to be higher in comparison to breast-specific gamma imaging (83.9% to 76.8%), while their specificity was comparable, with 58.8% and 70.6%, respectively [61]. The same results were acquired in the study by Lee et al., where the detectability rate of MRI for breast cancer recurrence was considerably higher in contrast to ultrasound imaging [62]. However, in another study to investigate the detection efficacy of MRI in breast cancer patients for axillary lymph node metastasis, post-neoadjuvant chemotherapy, its sensitivity, specificity, and positive and negative predictive value fell short compared to ultrasound imaging [63]. Moreover, the role of PET/CT for the expectation of pathologic reaction is not predominant in MRI [64]. Therefore, there is a tendency to use MRI.

Chu et al. conducted a meta-analysis to determine the diagnostic efficiency of DWI in breast cancer to monitor pathological responses to neoadjuvant chemotherapy. In this study, the pooled weighted values had sensitivity of 0.88, specificity of 0.79, a positive likelihood ratio of 4.1, a negative likelihood ratio of 0.16, and a diagnostic odds ratio of 26. The area under the receiver operator characteristic curve was 0.91. In the subgroup analysis, the pooled specificity of change in the ADC subgroup was higher than that in the pretreatment ADC subgroup [65]. The correlation of ADC and FA (fractional anisotropy) was also examined by others, and the results indicated that the ADC and FA values of malignant lesions were statistically lower than those of benign lesions, and the combined DTI and FT (fiber tractography) sensitivity, specificity, and accuracy were comparable to DCE-MRI (96%, 90%, and 94%, respectively) [66,67].

To direct treatment decisions, precise loco-regional staging and preoperative evaluation of breast cancer are considered crucial [68]. In 2021, Hashem et al. assessed the role of DCE and DWI in the preoperative staging of breast cancer. This study showed that DCE could detect the ductal carcinoma in situ (DCIS) component of the malignant lesions and give better tumor size and hence superior staging outcomes over sono-mammography. Moreover, they stated that qualitative DWI could be accompanied by ultrasonography to better assess the axillary nodal status. Both combinations enable the preoperative loco-regional staging of malignant breast disease to be effective and precise [69].

Ergul et al. compared the utility of 18F-fluorodeoxyglucose positron emission tomography/computed tomography (FDG PET/CT), DCE, and DWI and sentinel lymph node biopsy (SNB) in the diagnosis of axillary metastatic lymph nodes in early-stage breast cancer (ESBC) patients. According to these researchers, DCE-MRI provides relatively higher sensitivity than FDG PET/CT when investigating ESBC multifocality. They also suggested that FDG PET/CT has higher sensitivity and reliability than both DCE-MRI and DWI, and FDG PET/CT data will direct a selective SNB and axillary lymph node dissection (ALND) scheduling decision [70].

## 6. DTI in the Diagnosis of Breast Cancer

When diffusion in specific directions is more than in other directions, it is called anisotropic. In anisotropic diffusion, we cannot use a single diffusion coefficient; instead, we need to use a diffusion tensor.


$$D\;=\;[\begin{array}{ccc} D_{\mathrm{xx}} & D_{\mathrm{xy}} & D_{\mathrm{xz}}\\ D_{\mathrm{yx}} & D_{\mathrm{yy}} & D_{\mathrm{yz}}\\ D_{\mathrm{zx}} & D_{\mathrm{zy}} & D_{\mathrm{zz}} \end{array}]$$

DTI assesses water diffusion in a minimum of six directions. Parameters of DTI are ADC or mean diffusivity (MD), fractional anisotropy (FA), mean axial diffusivity λ_1_, mean radial diffusivity [(λ_2_ + λ_3_)/2], and empirical difference [λ_1_ − λ_3_].

The equation of ADC or MD is written as follows:


$$\frac{\lambda_{1}+\lambda_{2}+{\;\lambda }_{3}}{3}$$

FA is given by:


$$\frac{\sqrt{3}}{\sqrt{2}}\;\times \;\frac{\sqrt{{\left(\lambda_{1}-\mathrm{ADC}\right)}^{2}+{\left(\lambda_{2}-\mathrm{ADC}\right)}^{2}+{\left(\lambda_{3}-\mathrm{ADC}\right)}^{2}}}{\sqrt{\left(\lambda_{1}{}^{2}+\lambda_{2}{}^{2}+\lambda_{3}{}^{2}\right)}}$$

Comparing DWI with DCE shows that DWI is faster than DCE [3]. Moreover, DCE has false-positive results and unnecessary biopsies. Other studies have shown that DWI will enhance the accuracy of breast cancer diagnosis and characterization. On the other hand, DTI is an improvement of conventional DWI that quantifies anisotropy by evaluating water motion in six or more directions in addition to ADC [29,71,72,73]. Consequently, ADC is an essential diffusion factor for determining the difference between benign and malignant breast tumors (lighter on DWIs and darker on ADC maps relative to standard fibro-glandular tissue). At the same time, anisotropy measures can help further characterize the microstructure and microenvironment of the tumor [74].

Tsougos et al. evaluated DTI for differentiating breast tumors by setting b values to 0 and 600 s/mm^2^. The mean ADC value of malignant and benign lesions was 1.06 × 10^−3^ ± 0.24 mm^2^/s and 1.54 × 10^−3^ ± 0.22 mm^2^/s respectively, whereas it was 1.77 × 10^−3^ ± 0.20 mm^2^/s for normal tissue. They reported that ADC measurements had lower malignant lesions values than the benign and normal breast [4].

Changes in the DTI parameters were evaluated by Scaranelo et al. [75] before and after Gd (gadolinium) administration. They observed all breast cancers in the DDC (distributed diffusion coefficient) λ_1_ maps before and after administration of Gd-based contrast agents (GBCAs). The mean size of cancer extracted from λ_1_ maps before administration of GBCAs remained statistically indistinguishable from the size determined following administration. The cancers showed remarkably lower DDCs, mean diffusivity, and intensity after GBCA administration and no alteration in maximal anisotropy in comparison with prior GBCA administration. For all parameters, except for λ_3_, the mean AUC values before and after GBCA administration did not vary considerably [75].

Nissan et al. (2020) [76] compared the influence of lactation on breast cancer conspicuity using DCE with DTI parametric maps. They showed a reduction in the contrast-to-noise ratio of breast cancer to DCE values compared to non-lactating patients. DTI parameters of λ_1_, λ_2_, λ_3_, mean diffusivity, and λ_1_–λ_3_ significantly declined among lactating patients. Additionally, FA significantly increased in breast cancer associated with pregnancy compared to the normal lactating parenchyma region of interest. The contrast-to-noise in eigenvalues (λ_1_, λ_2_, λ_3_) and mean diffusivity were substantially superior to DCE in the lactating cohort [76].

The diagnostic performance of DTI parameters is controversial in the literature. In one study by Onaygil et al., breast cancer diagnostic factors were assessed. According to their results, in addition to the ability of this method to identify malignant breast lesions, DTI increased the sensitivity of the traditional 3 T breast MRI system and showed a correlation with estrogen receptor (ER) and Ki-67 biomarkers. However, in another study conducted by Abdelhady et al., the sensitivity and specificity of DTI in differentiating benign breast lesions from malignant tissue were lower than those of DWI [77]. Overall, DTI was proved to be a potential instrument for differential diagnosis to aid DCE-MRI and assess molecular subtypes in breast cancer [78].

## 7. DTI in Treatment Evaluation of Breast Cancer

The potential of DTI and DCE to monitor response to neoadjuvant chemotherapy was assessed by Edna Furman-Haran et al. [79]. In their study, the cancers scanned before neo-adjuvant chemotherapy in the three directional diffusion coefficients, λ_1_, λ_2_, and λ_3_, and the mean diffusivity. They found that the maximal anisotropy and l1–l3 had lower levels in cancerous locations than normal tissue, which were reported in other studies, as well [80,81]. They also reported an increase in the eigenvalues and mean diffusivity in response to neoadjuvant chemotherapy. Indeed, they showed that DTI can monitor alterations in the size and diffusion tensor parameters of breast cancer in response to neoadjuvant chemotherapy with an accuracy comparable to that of DCE [79].

Wilmes et al. investigated the prognostic importance of tumor metrics obtained from DTI. In this study, DTI and CE-MRI were carried out prior to treatment and after three cycles of taxane-based therapy (early treatment). According to the obtained results of tumor pretreatment, ADC was notably lower in the complete pathological response (pCR) than in the non-pCR group. At initial treatment, patients with PCR had a considerably more significant change in tumor eigenvalues (λ_1_, λ_2_, λ_3_) and ADC than those without pCR. Moreover, they observed that while there was a weak correlation in early percentage changes in tumor FA with pCR, the correlation with the final improvement in tumor volume with pCR is related to therapy [82].

Numerous studies have mentioned the ability of neurotoxicity of chemotherapy for breast cancer. For instance, Menning et al. assessed chemotherapy’s adverse effects on white matter. They examined this issue before and six months after chemotherapy, using matched intervals for the unexposed groups. Based on voxel analysis, their study did not indicate an effective chemotherapy with and without endocrine treatment on the integrity of white matter. Region of interest evaluation showed chemotherapy’s adverse effects with and without endocrine therapy by establishing a more significant decline in white matter (WM) integrity in the superior longitudinal fasciculus and corticospinal tract in breast cancer patients receiving systemic treatment than breast cancer patients who did not need chemotherapy [83].

## 8. Amide Proton Transfer-Weighted Imaging in Breast Cancer Diagnosis

As a molecular imaging technique, amide proton transfer-weighted imaging (APTWI) measures the concentration of unbound proteins and polypeptides in tissue with no need for extrinsic contrast agents. Instead of relying on water molecule diffusion within tissues, APTWI’s capacity to represent lesion information is achieved by monitoring the chemical rate of exchange between water and amide protons [84]. A study on the application of APTWI to assess breast lesions recently commenced. While several research findings have evaluated the utility of APTWI in tumor grading, cell proliferation [85], and its importance in treatment-related lymphedema therapy [86], few studies have examined APTWI’s effectiveness in diagnosing benign and malignant lesions and the connection among its parameters and prognostic factors [87]. Meng et al. attempted to evaluate the roles of diffusion kurtosis imaging (DKI) and APTWI in distinguishing benign and malignant breast lesions and examine the correlation coefficients between the obtained parameters and breast cancer prognostic factors to develop novel concepts for breast cancer diagnosis, treatment, and prognostic assessment. The results indicated that the DKI and APTWI both offer useful information about the characteristics of breast lesions, apparent kurtosis coefficient (Kapp), non-Gaussian diffusion coefficient (Dapp), and magnetization transfer ratio asymmetry (MTRasym (3.5 ppm)), which are all viable variables for determining the microstructure of tissue, and overall, DKI was found to be superior to APTWI in discriminating benign from malignant breast lesions for three main reasons. Firstly, the discrepancies in water molecule diffusion in cancer cells are larger than the protein and polypeptide composition changes. Secondly, the key contributors to alterations in the protein and polypeptide content of the microenvironment remain unknown. Finally, the APTWI technique is still being developed, and scanning precision could be improved [88].

## 9. Diffusion Kurtosis Imaging in Breast Cancer Diagnosis

While pure liquids and gels exhibit a Gaussian distribution of diffusion, obstacles induced by complicated tissue constructs effectively alter the probability distribution of diffusion. Kurtosis is the statistical term for assessing the true probability distribution within tissue. By obtaining supplementary images with a higher b-value (where b is an operator-defined parameter corresponding to the strength and duration of diffusion in imaged tissues), on the order of b = 1000–3000 s/mm^2^ and at least 15 diffusion gradient directions, the diffusion kurtosis imaging method can trace numerous structures inside a single voxel, for example, crossing white matter fibers in the brain. In the case of breast imaging, diffusion kurtosis imaging is susceptible to intracellular structures such as membranes and organelles [61] and can offer a diffusion heterogeneity index sensitive to tumor microstructure in addition to a mean kurtosis map [62]. Notably, when the unsuppressed fat signal is corrected for, diffusion kurtosis analysis of the breast improves [63].

Diffusion kurtosis imaging (DKI), proposed by Jenson et al., is a beneficial diffusion imaging technique [89]. Compared to standard DWI, DKI considers the non-Gaussian diffusion of water molecules within tissues and incorporates a fourth-order three-dimensional tensor into the structure of its model; as a result, DKI can approximate the microstructural heterogeneity of tissues [90]. Several researchers have explored the correlation between relevant DKI parameters and prognostic factors such as heterogeneous malignancy, discrepancies in pathological characteristics and grade, expression of ER and PR, and tumor diameter [57,91], but no conclusive findings were attained.

## 10. Magnetic Resonance Spectroscopy

Magnetic resonance spectroscopy (MRS) is a noninvasive imaging modality often implemented to assess the metabolic data inside a targeted tissue by revealing the spike in specific metabolites, such as total choline (tCho). Increased tCho levels have been identified in malignant tumors, including breast cancer, attributed to the increased cell membrane turnover of neoplastic processes. Numerous studies have shown that incorporating MRS into routine breast MR examinations enhances diagnostic performance and decreases the probability of needless biopsies. MRS has sufficient sensitivity of 71–74% and specificity of 78–88% for differentiating benign from malignant lesions [92,93,94]. Numerous findings have established a link between raised tCho concentrations and biologically aggressive cancer characteristics attributed to high grade, large dimensions, and a high Ki67 proliferation rate [95]. In addition to tCho, MRS is often used to identify and detect other metabolites, such as lipids, as abnormalities in lipid metabolism have been linked to cancer development in recent years. Thakur et al. established the diagnostic and prognostic utility of MRS by indicating that quantitative in vivo MRS analysis of the lipid metabolism of breast lesions enabled the detection of malignancies and subtypes of breast cancer [96]. Incorporating the lipid analysis into the tCho peak in MRS to detect breast cancer showed higher sensitivity compared to the situation where only one of them was measured [97].

## 11. Perspectives (Future Directions)

This review provides a broad overview and comparison of various MR imaging methods, including DCE, DWI, and DTI, for breast cancer diagnosis. As mentioned earlier, MRI is more sensitive than mammography and sonography [42,43]. DCE has limitations compared to other MRI models, such as high cost and inability to use in patients with renal dysfunction. However, comparisons of DCE, DWI, and T_2_-weighted techniques have shown that the use of DCE and DWI in diagnosing breast cancer is beneficial [48,49,98], but T_2_-weighted MRI cannot be significantly helpful [51]. Indeed, DWI plus T_2_-weighted MRI are less sensitive than DCE [5]. In addition to the diagnostic context, DWI has been evaluated in therapy planning, such as the evaluation of tumor response to treatment.

Table 1 and Table 2 summarize the essential criteria of different MRI methods for breast cancer diagnosis. As shown in Table 1, ADC has the most critical diffusion parameter in differentiating between benign and malignant breast lesions, while anisotropy measures can help further characterize the tumor [74]. In malignant lesions, the eigenvalues (λ1, λ2, λ3) and mean diffusivity or ADC due to diffusion limit should be less than in benign lesions and normal breasts. Moreover, ADC in malignant lesions was lower than in benign lesions and normal breasts. It has also been shown that in lactating patients, the values of λ_1_, λ_2_, λ_3_, mean diffusivity, and λ1–λ3 are significantly reduced. Moreover, FA is significantly increased in breast cancer associated with pregnancy compared to normal lactating parenchyma [76]. DTI study in therapy has also shown that eigenvalues and mean diffusivity increase in response to neoadjuvant chemotherapy [79].

Many studies have confirmed that DCE can determine tumor size and can have better staging than sono-mammography. The combination of DWI and ultrasound can also be valuable and accurate for the preoperative loco-regional staging of malignant tumor lesions [98]. In addition to examining the effectiveness of DWI in breast cancer, the significance of DTI has been considered. A comparison of DTI and DCE in the evaluation of tumor response to treatment has shown that before treatment, the amount of ADC in patients with pCR is less than in non-pCR patients. In early treatment in patients with pCR, the most changes in eigenvalues and ADC compared to non-pCR patients are observed [82].

Currently, because of the diversity in MRI models and instruments, only a few of them are available in clinics. With the development in MR instruments and technology, it is predicted that new MRI systems and methods will achieve the most advancement. Although many MR instruments and techniques are available, all have their limitations. In the future, with the development of instruments and advances in MR imaging modalities, software, image processing, and health management, they will provide a new platform for cancer diagnosis. These developments would also allow clinicians to predict better treatment.

For medical students, radiologists, and researchers interested in cancer diagnosis and therapy, this review article could be helpful. The limitation of this article is that we could not access all newly published papers or cover every aspect of all recent findings on breast cancer diagnosis.

## 12. Conclusions

Dynamic contrast-enhanced MRI (DCE-MRI) has high sensitivity and changeable specificity for breast cancer. DWI and its characteristics can show tumor cell thickness and microstructure or microvasculature at the cellular level without contrast agents. Recent findings have confirmed that DTI as an MR imaging modality has the potential for differential breast cancer diagnosis and could aid DCE-MRI and assess molecular subtypes in breast cancer detection. The diagnostic performance of new advances may be helpful in DTI, DWI, and DCE-MRI parameters and can help in the diagnosis of breast cancer in the early stages. Overall, recent findings showed that DWI and DTI and their characteristics may facilitate earlier and more accurate breast cancer diagnosis and treatment.

## Figures and Tables

**Table 1:** The most important DTI characteristics for breast cancer.

Ref.	λ_1_	λ_2_	λ_3_	MD	FA	λ_1_–λ_3_
Noam Nissan et al. [76] in pregnancy-associated breast cancer	1.17 ± 0.11	0.95 ± 0.11	0.74 ± 0.11	0.95 ± 0.11	0.25 ± 0.05±	0.43 ± 0.07
Haran et al. [79] (median % change responders)	55.7 (43.6–77)	55.4 (42.3–74.2)	61.5 (41.3–81.0)	55.6 (42.4–71.8)	1.3 (214.3–20.8)	55.4 (42.4–100.1)
Onaygil et al. [78]	1.91 ± 0.30 * 1.27 ± 0.19 **	1.68 ± 0.28 * 1.01 ± 0.20 **	1.46 ± 0.27 * 0.81 ± 0.24 **	1.68 ± 0.27 * 1.03 ± 0.19 **	0.14 ± 0.05 * 0.24 ± 0.14 **	0.45 ± 0.17 * 0.48 ± 0.25 **

λ_1_, λ_2_, λ_3_: eigenvalues, MD: mean diffusivity, FA: fractional anisotropy, λ_1_–λ_3_: empirical difference, ADC: apparent diffusion coefficient, *: benign, **: malignant.

**Table 2:** The most important DWI characteristics for breast cancer.

Ref.	(ADC: ×10^3^ mm^2^/s) Malignant	(ADC: ×10^3^ mm^2^/s) Benign
Egnell et al. [52] b-values (0, 200, 600, 1200, 1800, 2400, 3000) s/mm^2^	=1.04 (0.96–1.20)	=1.75 (1.51–1.86)
Pereira et al. [3] b-values (0, 250, 500, 750, and 1000)	0.907	1.45
Sinha et al. [29]	1.36 ± 0.36	2.01 ± 0.46

λ_1_, λ_2_, λ_3_: eigenvalues, MD: mean diffusivity, FA: fractional anisotropy, λ_1_–λ_3_: empirical difference, ADC: apparent diffusion coefficient.

## Data Availability

The data presented in this study are available on request from the corresponding author.
